# Brief report: Artificial intelligence meets small cell lung cancer—integrating clinicopathological and wholeslide image data for prognostic prediction in SCLC

**DOI:** 10.3389/frai.2026.1766576

**Published:** 2026-04-20

**Authors:** Pedro Rocha, Joan Gibert, Silvía Menendez, Raúl del Rey-Vergara, Albert Iñañez, Laura Masfarré, Nil Navarro, Alejandro Ríos-Hoyo, Sandra Perez, Álvaro Taus, Mario Giner, Ana Rovira, Luis León-Mateos, Dolores Isla, Luis Paz-Ares, Jon Zugazagoitia, Cristina Martí Blanco, Rosario García-Campelo, Alberto Moreno-Vega, Ángel Callejo, Federico Rojo, Ignacio Sanchéz, Edurne Arriola

**Affiliations:** 1 Department of Medical Oncology, Hospital del Mar, Barcelona, Spain; 2 Cancer Research Program, Hospital del Mar Research Institute, Barcelona, Spain; 3 Department of Pathology, Hospital del Mar, Barcelona, Spain; 4 Department of Pathology, IIS-Fundación Jiménez Díaz-CIBERONC, Madrid, Spain; 5 Centro de Investigación Biomédica en Red de Cáncer (CIBERONC), Madrid, Spain; 6 Department of Medical Oncology, Hospital Clínico Universitario and Instituto de Investigación Sanitaria de Santiago de Compostela (IDIS), Santiago de Compostela, Spain; 7 Department of Medical Oncology, Hospital Universitario Lozano Blesa, IIS Aragón, Zaragoza, Spain; 8 Department of Medical Oncology, Hospital Universitario 12 de Octubre, Madrid, Spain; 9 Centro de Investigación Biomédica en Red de Oncología, CIBERONC-ISCIII, Madrid, Spain; 10 Medical Oncology, Hospital Universitario Sant Joan de Reus, Reus, Spain; 11 Department of Medical Oncology, Health Research Institute, Hospital Universitario A Coruña, A Coruña, Spain; 12 Medical Oncology, Hospital Universitario Reina Sofía, Córdoba, Spain; 13 AstraZeneca Farmacéutica Spain S.A., Madrid, Spain

**Keywords:** artificial intelligence, biomarkers, deep learning, prognostic factors, small cell lung cancer

## Abstract

**Introduction:**

Small-cell lung cancer (SCLC) represents a unique clinical challenge characterized by its aggressive nature, poor prognosis, and limited therapeutic options. Upfront prediction of survival outcomes in this disease could impact patient care by refining risk stratification and thus, personalizing treatment strategies. Here, we investigate the utility of a deep learning (DL) model using digital pathology to predict outcomes of patients diagnosed with SCLC.

**Methods:**

We built a random forest (RF) model using clinical data and a DL based model using whole-slide image (WSI) as inputs from a total of 307 patients diagnosed with SCLC, including a training set of 263 patients, and a validation set comprising 44 patients who participated in the CANTABRICO phase IIIB clinical trial. Model performance was assessed using the area under the receiver operating characteristic curve (AUC) with 5-fold crossvalidation to minimize bias and variance of the performance. We report the mean and 95% confidence interval of the AUC values across the folds.

**Results:**

In the training set, the RF model achieved an AUC of 0.728 (95% CI: 0.662–0.792) for long-term overall survival (LT_OS) prediction, while the combined RF and DL model achieved an AUC of 0.744 (95% CI: 0.680–0.807). For long-term progression-free survival (LT_PFS) prediction, the RF model achieved an AUC of 0.689 (95% CI: 0.625–0.753), whereas the combined model achieved an AUC of 0.704 (95% CI: 0.640–0.767). Application of the combined RF and DL model to the validation cohort yielded an AUC for LT_OS of 0.604 (95% CI: 0.582–0.626) and an AUC for LT_PFS 0.690 (95% CI: 0.643–0.738), indicating potential clinical applicability.

**Conclusion:**

Our results showcase the feasibility of integrating clinicopathological data with WSI through a deep learning model to predict outcomes in patients with SCLC. This approach holds promise in helping physicians to personalize treatment strategies that better suit individual patient needs.

## Introduction

Small-cell lung cancer (SCLC) is an aggressive malignancy characterized by rapid tumor progression, high metastatic potential, and limited therapeutic options (Megyesfalvi et al., 2023; Solta et al., 2024). Despite recent advancements with the introduction of immune checkpoint inhibitors (Horn et al., 2018; Paz-Ares et al., 2019) and bispecific T-cell Engagers (BiTEs) (Ahn et al., 2023; Dowlati et al., 2024; Paz-Ares et al., 2023), SCLC remains a challenging disease with poor prognosis, highlighting the urgent need for improved risk stratification and personalized treatment approaches. Unlike non-small cell lung cancer, where molecular biomarkers guide therapeutic decisions, SCLC lacks established predictive or prognostic biomarkers, leaving clinical decisions dependent on traditional factors such as performance status and disease stage (Sen et al., 2024).

Artificial intelligence (AI), particularly deep learning (DL) models, have demonstrated significant potential in oncology by extracting high-dimensional data from medical images to enhance diagnostic and prognostic predictions (Kather et al., 2020; Shah et al., 2021). Digital pathology, utilizing whole-slide imaging (WSI) combined with AI-driven models, has shown promise in refining disease classification and outcome prediction in various cancers (Saillard et al., 2023; Shamai et al., 2022) including SCLC (Shibaki et al., 2024).

From a pathology perspective, SCLC is characterized by small, round cells arranged in a compact pattern, exhibiting a high proliferation rate with frequent mitotic figures along with neuroendocrine features. These distinct morphological characteristics make SCLC a strong candidate for exploring DL models using WSI. Here, we present a multimodal ensemble model that combines a DL-based WSI analysis pipeline with a random forest (RF) classifier incorporating clinical data, in order to predict long-term survival outcomes in patients diagnosed with SCLC.

## Materials and methods

### Patients

Our study included a total of 307 patients diagnosed with small cell lung cancer (SCLC), for whom comprehensive clinicopathological and follow-up data were available. Collected variables included age, clinical stage, treatment type, performance status, progression-free survival (PFS), and overall survival (OS). Whole Slide Images (WSIs) obtained at the time of diagnosis were also collected for all patients. Pathological specimens were obtained from multiple anatomical sites according to clinical indication and availability, including primary lung tumors and metastatic locations such as lymph nodes and liver. Only specimens containing ≥50% viable tumor cells and without significant crush artifacts were considered adequate and included in the analysis. The cohort comprised two datasets: a training set of 263 patients diagnosed at Hospital del Mar, Barcelona, Spain, and a validation set comprising 44 patients who were part of the CANTABRICO trial (*NCT04712903*)—a phase IIIb study of durvalumab plus platinum–etoposide in first-line treatment of extensive-stage small cell lung cancer that include patients with a diagnosed of SCLC that underwent first-line treatment with platinum based chemotherapy plus durvalumab. This study was approved by the Institutional Review Board at Hospital del Mar, with IRB number 2017/7174/I.

For survival analyses, long-term overall survival (LT_OS) was defined as OS exceeding 12 months in patients with extensive-stage SCLC and 25 months in patients with limited-stage SCLC. Long-term progression-free survival (LT_PFS) was defined as PFS exceeding 5 months for patients with extensive-stage SCLC and 14.3 months for those with limited-stage SCLC. Cut-offs were based on historical data from landmark trials IMpower-133 for extensivestage SCLC (Horn et al., 2018), and in the CONVERT trial for limited-stage SCLC (Faivre-Finn et al., 2017).

### Whole-slide image acquisition and digitization

Glass slides (FFPE tissue sections; H&E stained) were digitized under brightfield illumination using two scanner ecosystems depending on cohort origin. A representative diagnostic H&E WSI per patient was selected after pathology review.

#### Training cohort

slides were digitized using Roche VENTANA whole-slide scanners as per the routine workflows of the contributing pathology laboratories. Scanning was performed at 20 × or 40 × equivalent magnification. For the Roche Digital Pathology Dx VENTANA DP 200 or VENTANA iScan HT systems, the scanners use a 20 × objective with the ability to scan at both.

20 × and 40×; high-resolution output corresponds to 0.465 μm/pixel (20×) and 0.25 μm/pixel (40×), with automatic tissue detection, barcode reading, and ICC color management. Images are stored in Roche BIF format and managed through the Roche uPath ecosystem in routine deployments.

#### Validation cohort (CANTABRICO trial)

Philips IntelliSite Ultra Fast Scanner (40×). H&E and IHC–stained slides were digitized using the Philips IntelliSite Ultra Fast Scanner at 40 × equivalent magnification, and the system provides continuous autofocus during scanning. Digital slides were stored in iSyntax format and accessed through the Philips Image Management System (IMS) for review and downstream analysis.

#### Quality control and harmonization

WSI quality control included verification of focus sharpness, tissue coverage, staining fidelity, and absence of scanning artifacts (e.g., out-of-focus regions, striping, folds). Scans failing QC were re-scanned when possible. Before deep-learning inference/training, standardized tiling and stain normalization were applied to reduce scanner/staining variability.

### Development of the prognostic model

**Figure 1:**
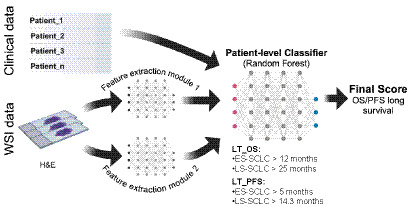
Overview of multimodal deep learning framework that integrates patient-specific data and histopathological images to predict clinical outcomes in patients diagnosed with small cell lung cancer. LT, Long-term; OS, Overall Survival; PFS, Progression-Free Survival; WSI, whole slide image.

Two predictive models were developed: a random forest (RF) model incorporating only clinical variables and a deep learning (DL) - based model using only WSIs as input. Additionally, a multimodal RF and DL model was constructed to integrate both clinical data and histopathological features extracted from WSIs, a detailed description of the model is provided in Supplementary methods S1. Model performance was assessed using the area under the curve (AUC), with 10-fold nested 5-fold cross validation applied to minimize bias and variance. The final model performance was reported as the mean AUC with a 95% confidence interval (CI) (Figure 1**)**.

### Preprocessing of the patch image dataset

WSIs obtained at diagnosis were preprocessed using a patch-based approach to extract relevant histopathological features. Presence of tumor tissue was confirmed by two pathologists (IS and MG). WSI processing and training was performed as previously described (Echle et al., 2020; Kather et al., 2020). Briefly, tissue segmentation was performed to exclude non-tumor regions, and each WSI was divided into smaller patches at a fixed magnification to capture detailed morphological features. To ensure consistency across slides, stain nor SHAP summary plots malization techniques were applied. Feature extraction was carried out using a convolutional neural network (CNN), allowing the identification of histopathological patterns associated with survival outcomes. The full description of the WSIs preprocessing pipeline can be found in Supplementary methods S2.

### Feature importance (multimodal patient-level classifier)

To interpret the multimodal patient-level classifier (clinical variables + WSI-derived features), we estimated global feature importance using a permutation-based approach implemented with the DALEX framework (explain_mlr3 + model_parts). For each predictor, we randomly permuted its values and quantified the resulting deterioration in model performance (“dropout loss”). We used B = 100 permutations to obtain a mean and standard deviation of the importance. To facilitate interpretability, dropout losses were rescaled to a relative importance percentage between the baseline and full model performance as:


$$\text{importance}(\%)=(1-\frac{\text{Lperm}-\text{Lbaseline}}{\text{Lfull}-\text{Lbaseline}})x100$$

where L_perm_ is the mean dropout loss for the permuted feature, L_baseline_ corresponds to the baseline loss and L_full_ to the full-model loss from model_parts.

We report importance for both outcomes (LT_OS and LT_PFS) and include WSI-derived features from the WSI model outputs used by the multimodal classifier. We use permutation based importance because impurity-based importance in tree models is known to be biased toward high-cardinality predictors.

### Statistical analysis

Descriptive statistics were used to summarize clinical and demographic characteristics. Differences between the training and validation cohorts were assessed using Chi-square tests for categorical variables and either t-tests or Mann–Whitney U tests for continuous variables, depending on their distribution.

To evaluate model performance, AUC values were calculated to assess discrimination ability. In order to assess the feature importance for each variable on model testing, *“predict parts”* function from the DALEX package was used. Kaplan–Meier survival curves were generated to visualize survival distributions across risk groups, and log-rank tests were performed to determine statistical significance between groups. Statistical analyses were conducted using R program 4.4.2.

## Results

### Clinical characteristics

A total of 307 patients diagnosed with small cell lung cancer (SCLC) were included in the study, with 263 patients assigned to the training cohort and 44 patients the validation cohort. The median age of patients in the training cohort was 67 years (range: 29–88), while in the validation cohort, the median age was 64.5 years (range: 51–84). Most patients were male, representing 78% of the training cohort and 64% of the validation cohort. Most patients had a history of smoking, with only a small proportion classified as never-smokers (2%). Regarding disease staging, limited-stage SCLC (LS-SCLC) accounted for 35% of patients in the training cohort, while all patients in the validation cohort had extensive-stage SCLC (ES-SCLC). ECOG performance status (PS) was predominantly 0–1 in both cohorts. Additionally, brain and liver metastases were present in 16 and 27% of the training cohort, respectively, and in 7 and 34% of the validation cohort (Table 1**)**.

**Table 1:** Clinicopathological characteristics of the population included in the training and validation cohort respectively.

Characteristics	Training cohort *N* = 263 (%)	Validation cohort *N* = 44 (%)
Age, median (range)	67 (29–88)	64.5 (51–84)
Sex		
Male	207 (78%)	28 (64%)
Female	56 (22%)	16 (36%)
Smoking status		
Never	5 (2%)	1 (2%)
Current/former	258 (98%)	43 (98%)
ECOG		
0–1	186 (71%)	42 (95%)
≥2–4	72 (27%)	2 (5%)
NA	5 (2%)	0 (0%)
Stage		
LS-SCLC	93 (35%)	0 (0%)
ES-SCLC	170 (65%)	44 (100%)
Brain M1		
Yes	43 (16%)	3 (7%)
No	163 (62%)	41 (93%)
NA	57 (22%)	0 (0%)
Liver M1		
Yes	70 (27%)	15 (34%)
No	180 (68%)	29 (66%)
NA	13 (5%)	0 (0%)

ECOG, Eastern Cooperative Oncology Group (ECOG) performance status; M1, metastases; NA, not available.

### Performance of the deep learning model

The random forest (RF) model trained on clinical data achieved an area under the curve (AUC) of 0.728 (95% CI: 0.662–0.792) for predicting long-term overall survival (LT_OS). When whole-slide image (WSI) data alone were used in a deep learning (DL) model, the AUC was 0.673 (95% CI: 0.596–0.744). Combining clinical and WSI data within a multimodal framework (RF + DL model) improved performance, achieving an AUC of 0.744 (95% CI: 0.680–0.807). For long-term progression-free survival (LT_PFS), the RF model based on clinical data achieved an AUC of 0.689 (95% CI: 0.625–0.753). The DL model using WSI data alone had lower performance, with an AUC of 0.501 (95% CI: 0.428–0.571). However, the combination of clinical and WSI data improved the AUC to 0.704 (95% CI: 0.640–0.767) (Table 2A and Supplementary results 1).

**Table 2:** Performance of our multimodal deep learning model in our (A) training and (B) validation cohort.

A							
Training cohort		Clinical data		WSI data		Clinical + WSI data	
	Groups	Model	AUC	Model	AUC	Model	AUC
LT_OS	Yes: 64; No: 199	RF	0.728 (0.662–0.792)	RN50	0.673 (0.596–0.744)	RN50 + RF	0.744(0.680–0.807)
LT_PFS	Yes: 117; No: 146	RF	0.689 (0.625–0.753)	RN50	0.501 (0.428–0.571)	RN50 + RF	0.704(0.640–0.767)

B					C	
Validation—Cantabrico cohort		Clinical + WSI data			Clinical and WSI data—LT_OS	
Models mean	Groups	Model	AUC		Variable	Importance score % (SD)
LT_OS	Yes: 14; No: 30	RN50 + RF	0.604 (0.582–0.626)		Age	9.35 (1.34)
LT_PFS	Yes: 30; No: 14	RN50 + RF	0.690 (0.643–0.738)		Feature 2 model 1	6.89 (1.49)
					Feature 2 model 2	7.54 (1.49)
					ECOG	31.8 (2.32)
					Stage	5.41 (0.999)
					Treatment received	16.3 (1.88)
					Feature 1 model 1	7.36 (2.70)
					Feature 1 model 2	7.48 (1.71)

Clinical and WSI data—LT_PFS	
Variable	Importance score % (SD)
Age	10.8 (2.44)
Feature 2 model 1	4.85 (3.23)
Feature 2 model 2	4.17 (2.38)
ECOG	25.8 (3.20)
Stage	23.7 (4.75)
Treatment received	30.9 (3.61)
Feature 1 model 1	4.41 (3.56)
Feature 1 model 2	3.76 (2.35)

(C) Interpretative importance score from the patient-level classifier. Feature 1 and 2 correspond to WSI-derived slide-level malignancy scores exported from two independent deep-learning slide-level classifiers trained using heterogeneous tumor cohorts. AUC, Area Under Curve; LT, Long-term; OS, Overall Survival; PFS, Progression-Free Survival; SD, standard deviation; WSI, whole slide image.

The predictive performance of the multimodal model was evaluated in the external validation cohort. For LT_OS prediction, the RF + DL model achieved an AUC of 0.604 (95% CI: 0.582–0.626). For LT_PFS, the model performed better, reaching an AUC of 0.690 (95% CI: 0.643–0.738), suggesting potential clinical applicability of the approach (Table 2B and Supplementary results 1). In this cohort, Kaplan–Meier survival analysis demonstrated a statistically significant difference between the predicted risk groups. The median overall survival (OS) was not reached in the group predicted to have longer survival, whereas it was.

7.31 months in the group predicted to have shorter survival (Supplementary Figure 3).

### Feature importance analysis

To further interpret the multimodal model, feature importance scores were derived from the patient-level classifier. In LT_OS prediction, age had the highest importance score (9.35 ± 1.34), followed by histopathological features extracted from the WSI-based model, illustrative 239 case examples can be found in Supplementary Figure 1. Other significant contributors 240 included ECOG performance status, disease stage, and treatment received. Similarly, for 241 LT_PFS prediction, age and WSI-derived features played a significant role, along with ECOG 242 performance status and disease stage (Table 2C and Supplementary Figure 2).

## Discussion

We developed and validated a DL model integrated with a RF classifier using clinical and histopathological data to predict long-term survival outcomes in SCLC patients. Our findings demonstrate that combining WSI with clinical data enhances prognostic accuracy compared to using either modality alone. These results highlight the potential of artificial intelligence driven approaches in improving risk stratification and predict survival outcomes in SCLC. Artificial intelligence (AI) has demonstrated transformative potential in non-small cell lung cancer (NSCLC). Deep learning models trained on whole-slide histopathology images can accurately classify adenocarcinoma subtypes and predicting spatial prognosis risk scores, enabling patient stratification into distinct prognostic groups (Ding et al., 2023). CT-based deep learning successfully classifies tumor invasiveness and predicts micropapillary patterns, facilitating preoperative risk stratification (Ding et al., 2020). AI has also enhanced clinical workflow efficiency in lung cancer management (Ding et al., 2025).

In contrast, SCLC lacks well-established predictive or prognostic biomarkers, leaving clinicians reliant on traditional factors such as ECOG performance status and disease stage, creating urgent need for AI-powered risk stratification and biomarker discovery in this underserved population.

Our study confirms the prognostic value of these factors, as age, ECOG status, and tumor stage were among the most important contributors to survival prediction in our model. However, integrating deep learning-based histopathological analysis provided additional predictive power, suggesting that morphological features captured from WSIs contain prognostic information that extends beyond conventional clinical parameters. Our results indicate that the standalone DL model using WSI features had a lower predictive performance (AUC of 0.673 for LT_OS and 0.501 for LT_PFS), highlighting the challenge of using image based models alone in SCLC prognosis. However, the combination of clinical and WSI-derived features within a multimodal framework significantly improved the predictive accuracy, achieving an AUC of 0.744 for LT_OS and 0.704 for LT_PFS in the training cohort. These findings suggest that while clinical variables remain fundamental in prognosis, AI-driven histopathological analysis provides complementary insights that enhance overall predictive performance. The external validation cohort confirmed the positive signal observed in the training cohort, with the multimodal model achieving an AUC of 0.604 for LT_OS and 0.690 for LT_PFS. Although model performance declined in the validation cohort, which may be attributed to differences in patient populations and treatment strategies between the training and validation datasets, the results still support the feasibility of integrating AI-driven histopathological analysis in clinical prognostic assessments.

Our AI-based prognostic model demonstrates performances advantages over traditional SCLC prognostic features such as TNM stage (Liang et al., 2022; Liu et al., 2023; Wang et al., 2018). While studies in SCLC remain scarce, pathology-based AI models in NSCLC have shown AUC of 0.80–0.96 for survival prediction, reflecting their ability to extract sub-visual morphological features and spatial patterns in the tumor microenvironment not discernible through conventional assessment (Li et al., 2025; Vo et al., 2025). The integration of AI into digital pathology has the potential to revolutionize prognostic modeling in SCLC by enabling objective, automated analysis of histopathological images. Given that WSIs are routinely collected in clinical practice, AI-driven models could be easily integrated into existing workflows to assist oncologists in risk stratification and prognosis. Moreover, the identification of prognostic histopathological patterns could pave the way for novel biomarker discovery in SCLC, an area where predictive markers are currently lacking. Future studies should focus on expanding dataset sizes to improve model robustness and explore the integration of molecular data with clinical and histopathological features. Additionally, prospective validation in clinical trials is necessary to determine the real-world applicability of these AI-driven approaches in guiding treatment decisions. Key barriers to adoption of these models include establishing pathologist trust through transparent validation, addressing subspecialty-specific needs, securing regulatory approval, and developing appropriate reimbursement models (Cheng et al., 2021; Reis-Filho and Kather, 2023).

Our study is not without limitations. First, the validation cohort was smaller and comprised exclusively of patients diagnosed with ES-SCLC, while the training cohort included 35% of patients diagnosed with LS-SCLC. This imbalance in stage distribution may have influenced the model’s generalizability and contributed to the reduced performance observed in the validation set. Second, while our WSI preprocessing pipeline applied standardized stain normalization techniques, variations in slide preparation across time, scanning and institutions could introduce potential biases. Additionally, our dataset includes samples from the primary tumor (lung) as well as specimens obtained from metastatic sites. Therefore, the potential impact of tissue background from different organs cannot be ruled out in our model and warrants further investigation in future studies. Finally, our study was retrospective, and prospective validation is required to confirm the clinical utility of our model in real-world settings.

In conclusion, our study demonstrates the feasibility of integrating clinical data with AI-driven histopathological analysis to predict survival outcomes in SCLC patients. The multimodal deep learning approach outperformed individual clinical or WSI-based models, highlighting the added value of digital pathology in prognostic modeling. Inclusion of additional biomarkers (i.e., immunohistochemical markers, genomic alterations) might further increase the potential of prediction using deep learning models. This approach holds promise in helping physicians in personalize treatment strategies that better suit individual patient needs.

## Data Availability

The original contributions presented in the study are included in the article/Supplementary material, further inquiries can be directed to the corresponding author.
