# Supplementary material for: Brief report: Artificial intelligence meets small cell lung cancer—integrating clinicopathological and wholeslide image data for prognostic prediction in SCLC
Source: Front Artif Intell. 2026 Apr 20;9:1766576. doi: 10.3389/frai.2026.1766576 (PMC13136249; doi:10.3389/frai.2026.1766576)
Supplement: Supplementary file 1 [file Data_sheet_1.docx]

# Supplementary Methods S1 – Deep learning architecture and training configuration

### S1.1 Deep learning architecture (WSI model).

We used a patch-based WSI pipeline adapted from the KatherLab Histopathology Image Analysis (HIA) framework (PyTorch). For the main WSI experiments reported in the manuscript, the backbone was a ResNet‑50 (RN50) convolutional neural network (torchvision implementation; ResNet v1.5).

- Stem: 7×7 convolution (64 channels, stride 2) + batch normalization + ReLU, followed by 3×3 max‑pooling (stride 2).
- Residual stages: four stages with bottleneck residual blocks in a 3–4–6–3 configuration (conv2_x…conv5_x), with an expansion factor of 4 (final stage output 2048 channels).
- Head: global average pooling and a fully connected classification layer producing two logits (“Yes”/“No”) for the binary outcome.
- Input: tiles of 512×512 pixels were resized to the network’s expected input size (224×224 for RN50).

RN50 was selected as a widely used and well‑validated backbone for histopathology image classification, offering a strong accuracy–compute trade‑off and compatibility with the HIA workflow.

### S1.2 Training configuration.

Hyperparameters were set through experiment configuration files (JSON) and were kept constant across outcomes unless stated otherwise. We used 5‑fold stratified cross‑validation at the patient level (k=5). In each fold, approximately 80% of patients were used for training and 20% were held out for testing; within the training fold, 10% of tiles were randomly sampled (stratified by class) and used as a validation set for monitoring/model selection. For each WSI, up to 512 tiles were randomly sampled per slide (maxNumBlocks=512) to standardize slide‑level sampling and control memory. For completeness, the main training hyperparameters for both RN50 and ViT models are summarized in Table S1 below.

| Outcome label | Backbone | Epochs | Batch size | Optimizer / LR | L2 reg (weight decay) |
| --- | --- | --- | --- | --- | --- |
| LT_OS (OS_Long) | ResNet-50 | 100 | 64 | adam / 0.0001 | 1e-05 |
| LT_PFS (RelapseDX) | ResNet-50 | 100 | 64 | adam / 0.0001 | 1e-05 |

All DL experiments used Adam optimization with learning rate 1e‑4 and L2 regularization (weight decay) 1e‑5. The loss function was cross‑entropy over two classes (“Yes”/“No”). Unless otherwise noted, the backbone was fully fine‑tuned (freezeRatio=0). No explicit data augmentation was applied beyond random tile sampling and resizing/conversion to tensor.

### S1.3 Multimodal integration with clinical random forest.

We trained: (i) a clinical-only random forest (RF), (ii) a WSI-only DL model, and (iii) a multimodal model where WSI-derived predictions were added as additional numeric input features to the clinical RF classifier. The exact clinical and WSI-derived predictors used in the RF and multimodal models are listed in Supplementary Methods S3.

### S1.4 Computational environment.

Computational environment. Training and inference were run on an on-premise GPU server. The experiments reported in this manuscript were executed using NVIDIA GeForce RTX 2080 Ti GPUs (11 GB). At the time of execution, nvidia-smi reported NVIDIA driver version 530.30.02 and CUDA driver API compatibility version 12.1 (this is the maximum CUDA version supported by the installed driver; it can differ from the CUDA runtime shipped with a given framework build). All experiments were run in a conda environment using Python 3.8.5, PyTorch 1.7.1, and torchvision 0.8.2.

# Supplementary Methods S2 – Stain normalization and tile extraction (updated)

All slides were diagnostic H&E specimens from confirmed SCLC cases. Prior to digitization and inclusion, the presence of tumour tissue and overall slide adequacy were confirmed by board‑certified pathologists. No manual region‑of‑interest (ROI) annotation was performed; tiling was applied to all tissue regions after automated tissue segmentation.

### S2.1 Tile extraction using Bio-Formats

Tiles were generated directly from WSIs using a Python script based on python-bioformats (Java Bio-Formats via javabridge). The script iterates over WSI files in the input folder (supported extensions include .svs, .tiff/.tif, .jpeg, .mrxs, .bif, .ndpi), opens each slide with a Bio-Formats image reader, selects the desired pyramid series ("level"), and extracts non-overlapping tiles on a regular grid.

Execution command used in this work (example for 40× slides):

***python generate_tiles_bioformats.py --wsi_path /path_to_Slides/ --out_path / path_to_Slides_Tiles_Level/ --size 512 --level 1 --only_list False***

For 40× slides, tiles were extracted at pyramid level 1 with tile size 512×512 pixels. For slides scanned at 20×, tiles were extracted at pyramid level 0 (same tile size), to keep comparable effective resolution.

### S2.2 Key extraction parameters and quality filters.

| Parameter | Value (this study) |
| --- | --- |
| Tile size | 512×512 pixels (command line: --size 512) |
| Effective magnification | ≈20× (20× slides at level 0; 40× slides at level 1) |
| Tile overlap / stride | Overlap=0 (default --overlap 0; stride = tile size) |
| Pyramid level | Level 1 for 40× slides; Level 0 for 20× slides |
| Tissue-content filter enabled | Yes (default --check_tissue True) |
| Tissue threshold | 0.65 (mean tissue mask fraction > threshold) |
| Tissue mask method | Otsu threshold on grayscale + morphological disk(5) dilation + hole filling; excludes near-empty/near-saturated tiles |
| Output format | PNG tiles saved per-slide folder; filenames encode level and coordinates |
| Optional coordinate-only mode | --only_list True writes coordinates CSV without saving PNGs |

### S2.3 Stain normalization

After tile extraction, stain normalization was applied at tile level using the Macenko method. A single target tile image was used as the reference to fit the normalizer; all tiles were then transformed to match this reference. As an additional guard against background tiles, Normalize.py computed a Canny edge-density heuristic (Canny 40/40) and only normalized tiles with edge density >2%.

### S2.4 Code availability.

The exact scripts used are provided as Supplementary Code C1 (Normalize.py) and Supplementary Code C2 (generate_tiles_bioformats.py). These scripts fully specify tile generation and stain normalization as used for the experiments reported in this manuscript.

# Supplementary Methods S3 – Detailed list of clinical and histopathological predictors

This supplement provides a detailed inventory of all predictors used in the clinical-only (RF) and multimodal (clinical + WSI-derived) models, including the number of predictors, their definitions, and how they were collected/derived together with the main software/tools used for extraction and preprocessing (TRIPOD+AI). The clinical-only RF used 4 clinical predictors; the multimodal RF used those 4 clinical predictors plus 4 WSI-derived probabilistic scores (8 predictors in total).

### S3.1 Clinical predictors (used in RF and multimodal models).

**Age:** Numeric (years at diagnosis). Extracted from institutional clinical database.

**Stage_bin:** Binary stage grouping derived from clinical stage at diagnosis: Stage <4 vs Stage=4 (as in the analysis script).

**Treatment_type:** Categorical treatment group (from clinical database; harmonized as Treatment_type).

**PS:** Performance status derived from ECOG (0/1/2 mapped to ordered factor Level_0/Level_1/Level_2).

### S3.2 Outcomes (targets).

**OS_long (LT_OS):** Binary label indicating long overall survival. Threshold is stage-dependent (as implemented in analysis script).

**PFS_long (LT_PFS):** Binary label indicating long progression-free survival (definition as in analysis script).

### S3.3 Histopathological predictors derived from WSIs (multimodal models).

Whole-slide image (WSI)-derived predictors are obtained from deep-learning slide-level classifiers trained on H&E WSIs. These models provide continuous slide-level scores (predicted probability of the positive class) summarizing the presence of malignant tumor morphology in a given slide. The multimodal patient-level model uses these WSI-derived scores as additional numeric predictors together with the clinical variables listed in Section S3.1.

To promote robust morphology learning rather than cohort-specific artifacts, the WSI slide-level classifiers were trained using a heterogeneous non-SCLC tumor types (e.g., lung adenocarcinoma and colorectal carcinoma, among others) as auxiliary comparators. This multi-tumor training strategy serves as a transfer/generalization mechanism.

Two independent WSI architectures were trained and evaluated (ResNet-50 and Vision Transformer). For each outcome (LT_OS and LT_PFS), the corresponding slide-level model outputs (one score per architecture per slide) were exported at patient level and used as inputs for the multimodal learner. For reproducibility, the exported prediction files are provided as supplementary data.

Table 2C uses generic labels (“Feature 1” and “Feature 2”, model 1/2) to keep the table readable. These features are not handcrafted histopathological measurements; they are WSI-derived slide-level class probabilities exported from the deep-learning models.

- Model 1 = ResNet‑50; Model 2 = Vision Transformer (ViT).
- Feature 1 = predicted probability of the positive class (“Yes”).
- Feature 2 = predicted probability of the negative class (“No”; 1 − Feature 1).
- For LT_OS, “Yes/No” probabilities are those exported from the WSI OS_long classifier.
- For LT_PFS, “Yes/No” probabilities are those exported from the WSI RelapseDX classifier.

### S3.4 Data extraction and software/tools.

**Tile extraction from WSI:** generate_tiles_bioformats.py (Bio-Formats backend) with tile size 512 px; pyramid level 1 for 40× and level 0 for 20× scans.

**Stain normalization:** Normalize.py (stain-normalization script used in preprocessing).

**WSI model training/inference:** Deep learning models (ResNet-50) trained on extracted tiles; slide-level probabilities exported as patient-level CSVs.

**Clinical and multimodal modeling:** R (mlr3 + ranger) with one-hot encoding for categorical predictors.

**Feature importance:** DALEX / DALEXtra model_parts permutation importance (B=100).
