# Supplementary material for: Brief report: Artificial intelligence meets small cell lung cancer—integrating clinicopathological and wholeslide image data for prognostic prediction in SCLC
Source: Front Artif Intell. 2026 Apr 20;9:1766576. doi: 10.3389/frai.2026.1766576 (PMC13136249; doi:10.3389/frai.2026.1766576)
Supplement: Supplementary file 2 [file Data_sheet_2.docx]

# Supplementary Results – Model Performance Visualization (ROC curves and confusion matrices)

**Supplementary results 1 ROC curves**

Figure S1. ROC curves for LT_OS (Internal cohort).


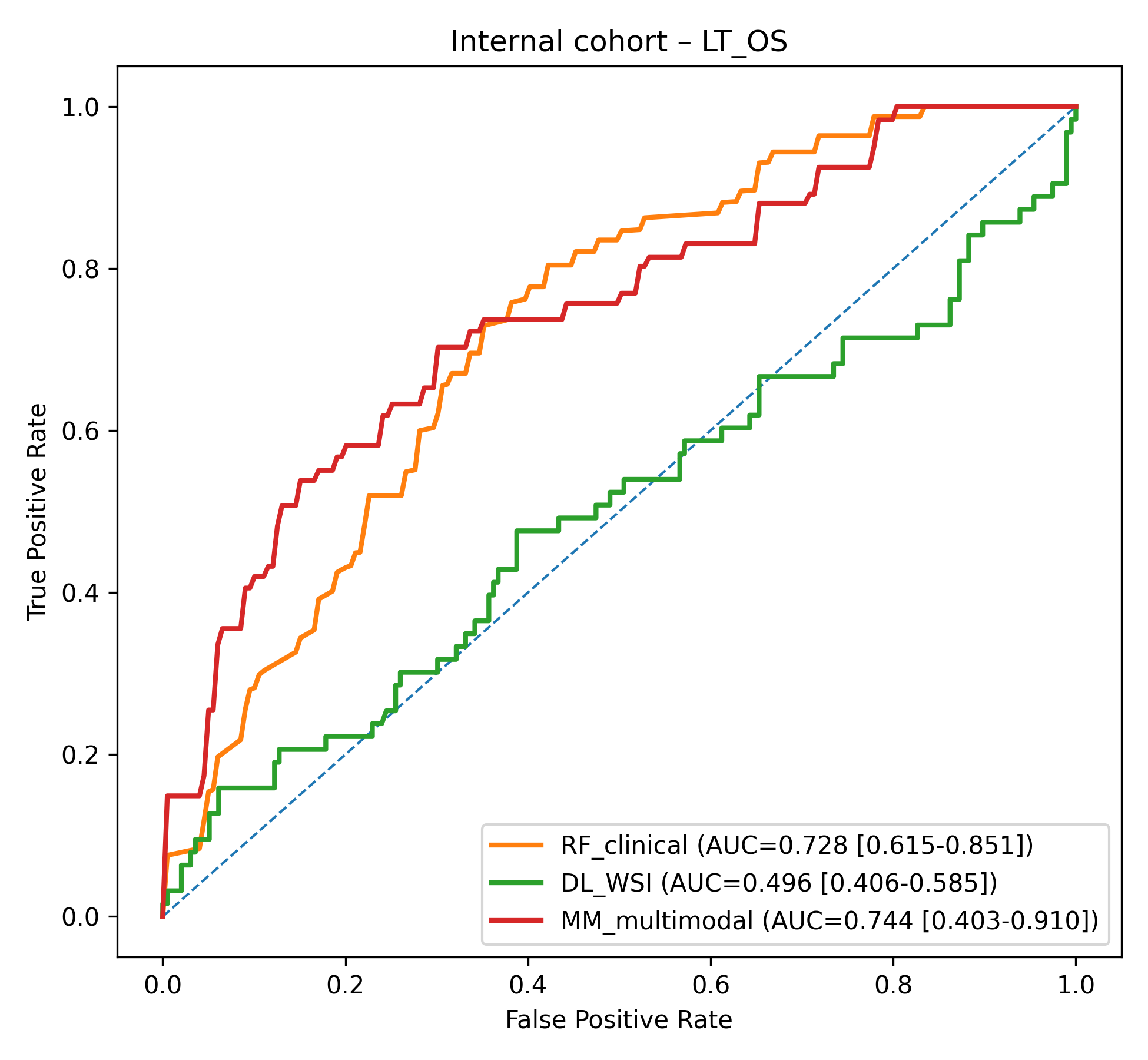


Figure S2. ROC curves for LT_PFS (Internal cohort).


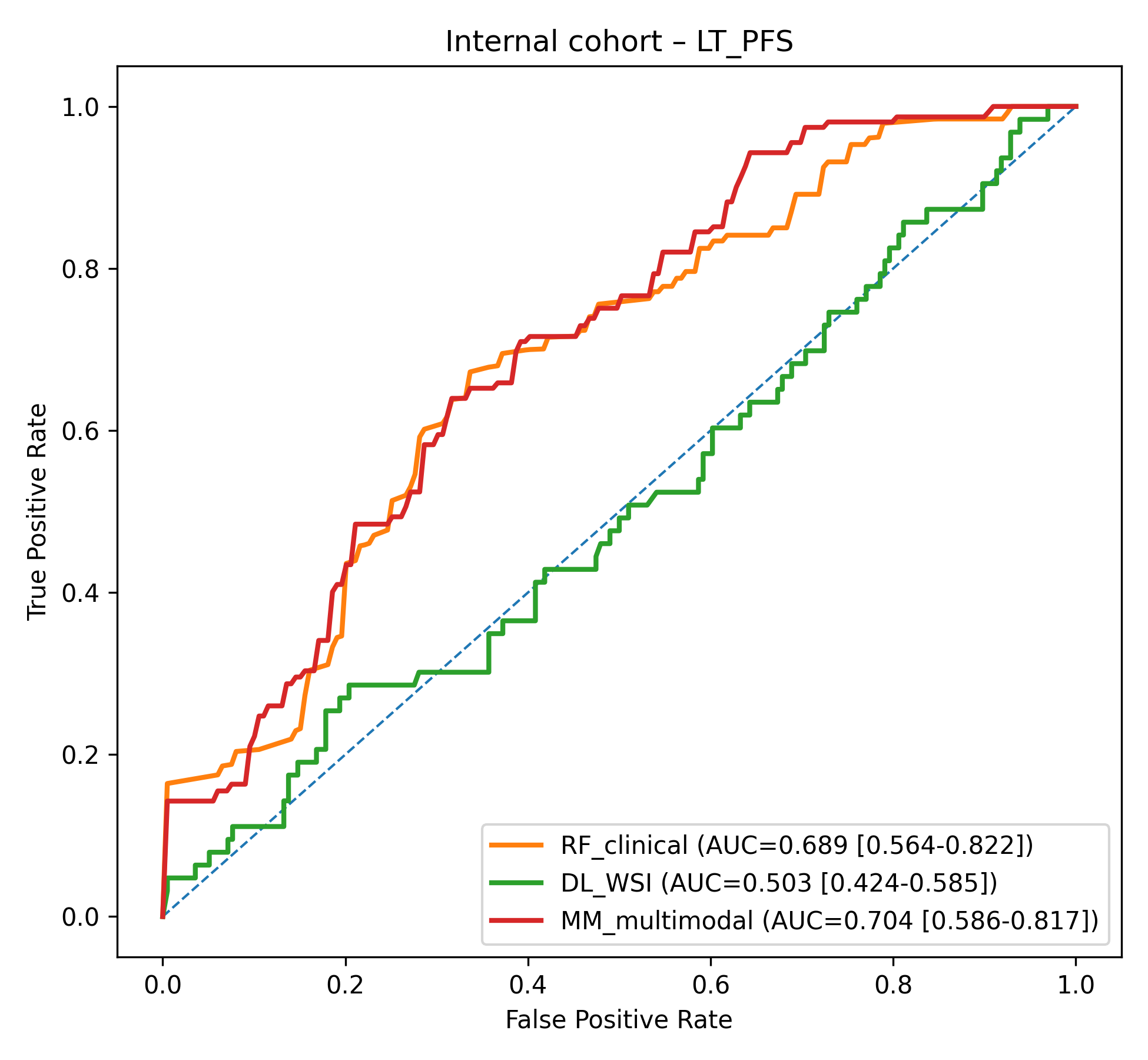


Figure S3. ROC curves for LT_OS (Cantabrico cohort).


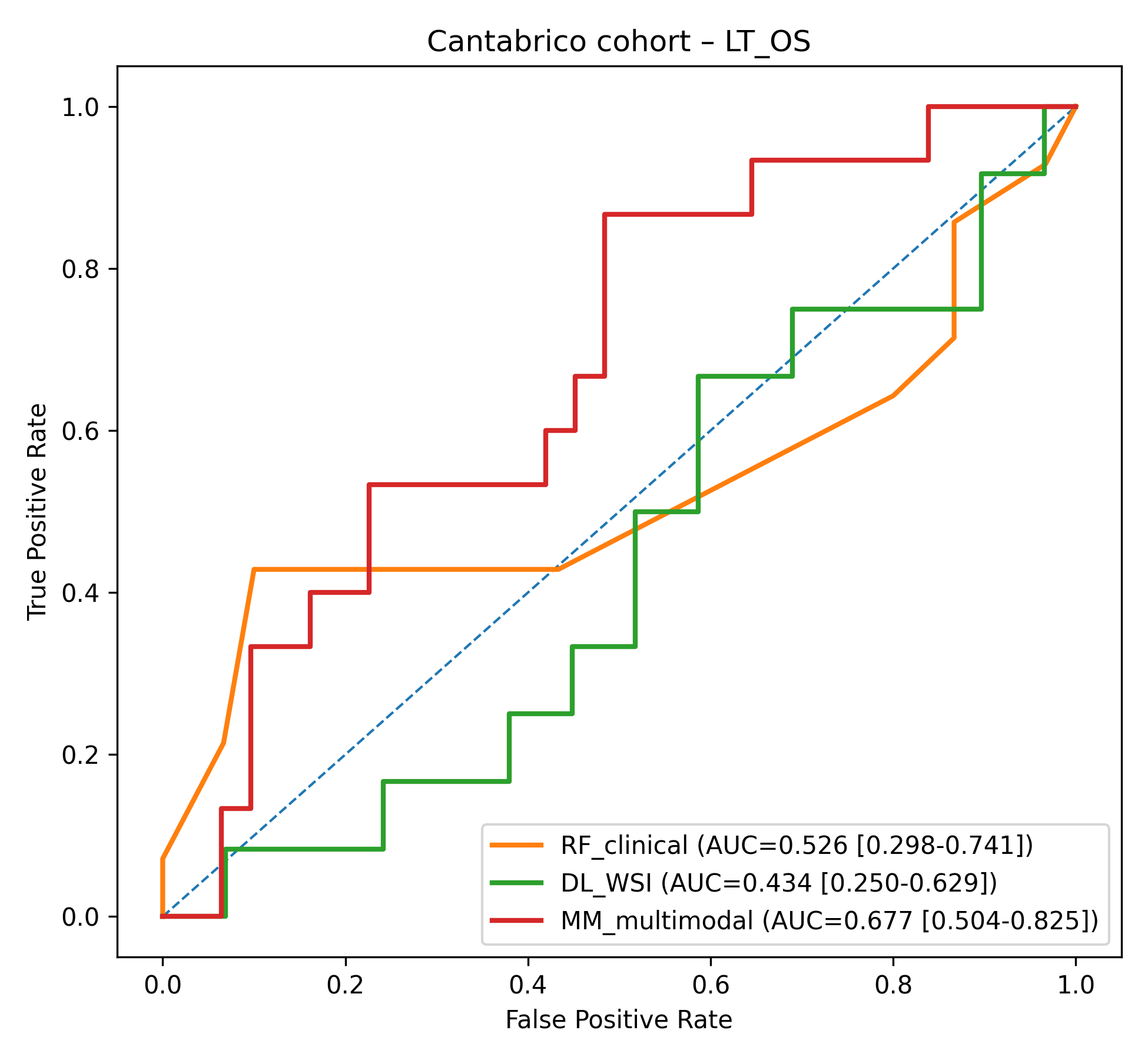


Figure S4. ROC curves for LT_PFS (Cantabrico cohort). Note: DL_WSI uses n=41 (WSI-available subset), whereas RF/MM use n=44.


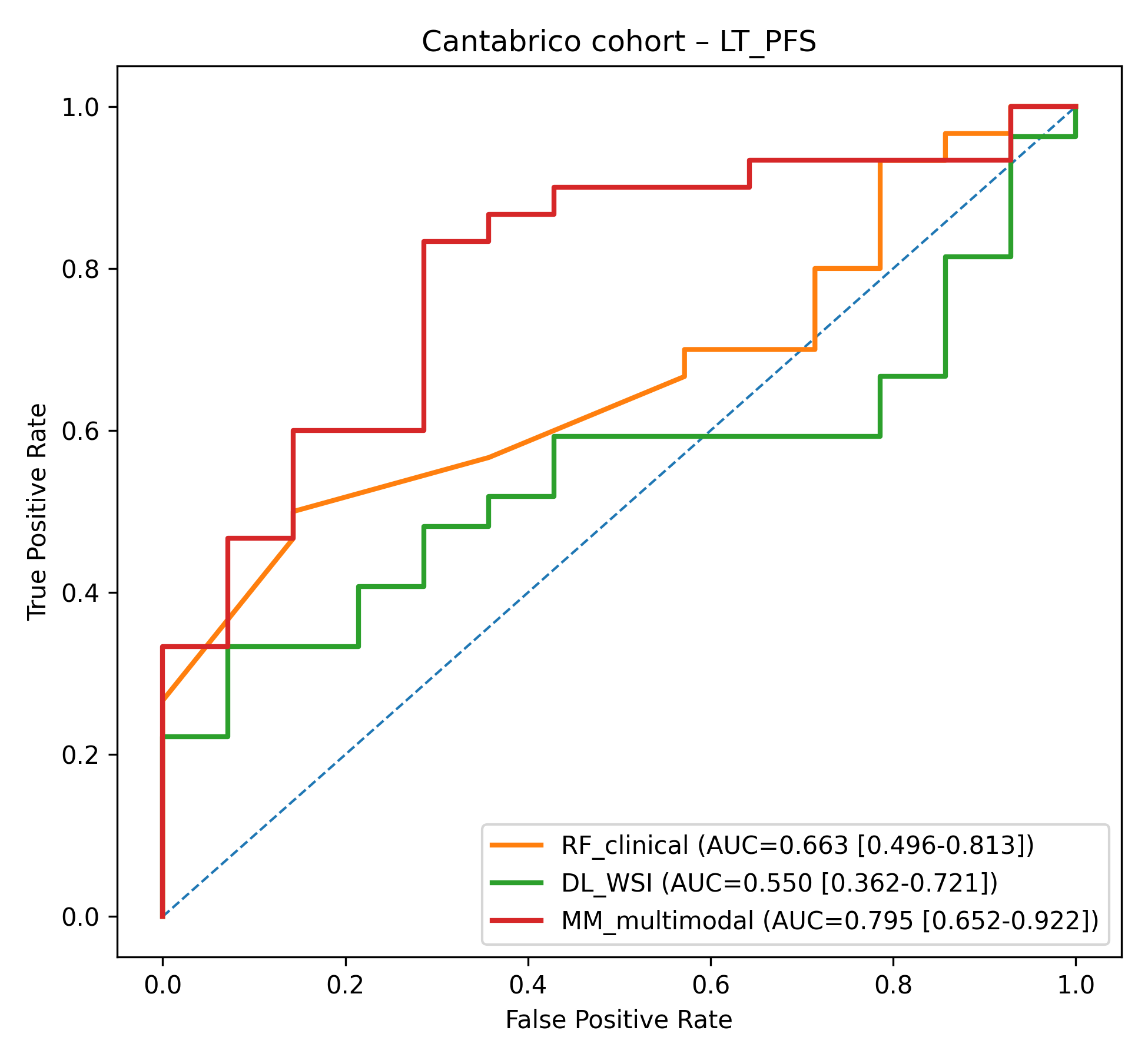


## Supplementary results 2. Metrics table (AUC and threshold-dependent metrics)

Table S1 summarizes AUC (with 95% confidence intervals estimated by non-parametric bootstrapping; 1,000 resamples) and threshold-based metrics at probability threshold 0.50 (accuracy, sensitivity, specificity, precision, F1 score).

| cohort | endpoint | model | auc | auc_ci_low | auc_ci_high | accuracy | sensitivity | specificity | precision | f1 |
| --- | --- | --- | --- | --- | --- | --- | --- | --- | --- | --- |
| Internal | LT_OS | DL_WSI | 0.496 | 0.406 | 0.585 | 0.544 | 0.476 | 0.566 | 0.261 | 0.337 |
| Internal | LT_OS | MM | 0.744 | 0.403 | 0.91 | 0.764 | 0.031 | 1.000 | 1.000 | 0.061 |
| Internal | LT_OS | RF | 0.728 | 0.615 | 0.851 | 0.757 | 0.000 | 1.000 |  | 0.000 |
| Internal | LT_PFS | DL_WSI | 0.503 | 0.422 | 0.579 | 0.448 | 0.571 | 0.408 | 0.237 | 0.335 |
| Internal | LT_PFS | MM | 0.704 | 0.586 | 0.817 | 0.620 | 0.359 | 0.829 | 0.627 | 0.457 |
| Internal | LT_PFS | RF | 0.689 | 0.564 | 0.822 | 0.631 | 0.513 | 0.726 | 0.600 | 0.553 |
| Cantabrico | LT_OS | DL_WSI | 0.434 | 0.25 | 0.629 | 0.488 | 0.250 | 0.586 | 0.200 | 0.222 |
| Cantabrico | LT_OS | MM | 0.677 | 0.504 | 0.825 | 0.682 | 0.000 | 1.000 |  | 0.000 |
| Cantabrico | LT_OS | RF | 0.526 | 0.298 | 0.741 | 0.682 | 0.000 | 1.000 |  | 0.000 |
| Cantabrico | LT_PFS | DL_WSI | 0.55 | 0.362 | 0.721 | 0.488 | 0.222 | 1.000 | 1.000 | 0.364 |
| Cantabrico | LT_PFS | MM | 0.795 | 0.652 | 0.922 | 0.591 | 0.433 | 0.929 | 0.929 | 0.591 |
| Cantabrico | LT_PFS | RF | 0.663 | 0.496 | 0.813 | 0.636 | 0.833 | 0.214 | 0.694 | 0.758 |
